# Modulatory role of vitamin A on the C*andida albicans*-induced immune response in human monocytes

**DOI:** 10.1007/s00430-014-0351-4

**Published:** 2014-08-17

**Authors:** Tilman E. Klassert, Anja Hanisch, Julia Bräuer, Esther Klaile, Kerstin A. Heyl, Michael M. Mansour, Jenny M. Tam, Jatin M. Vyas, Hortense Slevogt

**Affiliations:** 1 Septomics Research Center, Jena University Hospital, Albert-Einstein-Strasse 10, 07745 Jena, Germany; 2 Center for Sepsis Control and Care (CSCC), Jena University Hospital, Erlanger Allee 101, 07747 Jena, Germany; 3 Division of Infectious Diseases, Department of Medicine, Massachusetts General Hospital, Harvard Medical School, 55 Fruit Street, GRJ-5-504, Boston, MA USA

**Keywords:** Vitamin A, Retinoic acid, Candida albicans, Dectin-1

## Abstract

Beyond its well-documented role in reproduction, embryogenesis and maintenance of body tissues, vitamin A has attracted considerable attention due to its immunomodulatory effects on both the innate and the adaptive immune responses. In infectious diseases, vitamin A has been shown to have a host-protective effect in infections of bacterial, viral or protozoan origin. Nevertheless, its impact in fungal infections remains unknown. Meanwhile, the frequency of invasive mycoses keeps on growing, with *Candida albicans* being the major opportunistic fungal pathogen and associated with high mortality. In the present work, we explored the impact of all-trans retinoic acid (atRA), the most active metabolite of vitamin A, on the innate immune response against *C.* *albicans* in human monocytes. Our results show a strong immunomodulatory role for atRA, leading to a significant down-regulation of the fungi-induced expression and secretion of the pro-inflammatory cytokines TNFα, IL6 and IL12. Moreover, atRA significantly suppressed the expression of Dectin-1, a major fungal pattern recognition receptor, as well as the Dectin-1-dependent cytokine production. Both RAR-dependent and RAR-independent mechanisms seem to play a role in the atRA-mediated immunomodulation. Our findings open a new direction to elucidate the role of vitamin A on the immune function during fungal infections.

**Electronic supplementary material:**

The online version of this article (doi:10.1007/s00430-014-0351-4) contains supplementary material, which is available to authorized users.

## Introduction

Vitamin A is an essential nutrient obtained through the diet either as provitamin-A (carotenoids) or as preformed vitamin A (retinol and retinyl esters) [1, 2]. In its esterified form, it can be stored in the liver, where it is continually hydrolyzed to retinol and deployed into circulation [3, 4]. Once in its target tissues, two dehydrogenases are able to convert the retinol into retinoic acid, the biologically active metabolite of vitamin A [5]. In this form, vitamin A is known to play an essential role in multiple biological processes, including reproduction, embryogenesis, maintenance of body tissues and augmentation of the immune system [6–8]. Over the last decades, an increasing effort has been devoted to better define its involvement in the regulation of the immune response, since vitamin A deficiency (VAD) has been associated with an increased susceptibility to severe infectious diseases [9].

Several in vitro studies have shed light into the role of vitamin A not only as an important factor for normal immune system development, but also as a modulator of both the innate and the adaptive immune responses [4, 10]. Vitamin A has shown to regulate the development of B-lymphocytes and its immunoglobulin production [11–13]. In T-cells, retinoic acid is able to attenuate the Th1-associated gene expression and skew the immune response toward a Th2 profile [14]. Retinoic acid is also able to modulate the LPS-induced cytokine and/or chemokine production in further innate immune cells, including monocytes [15], macrophages and dendritic cells [16]. Moreover, the role of vitamin A as immunomodulator has been reinforced by interventional studies with infants and children with VAD. Here, vitamin A supplementation could reduce the morbidity and/or mortality from measles, malaria and certain forms of diarrhea [17]. These findings are also supported by several in vitro and animal studies, where a host-protective effect of vitamin A could be described for infections involving a bacterial, viral or protozoan origin [18]. Nevertheless, the ability of vitamin A to modulate the immune response against fungal infections is still unknown.

The frequency of invasive mycoses due to opportunistic fungal pathogens has been significantly growing in intensive care units over the last decades [19, 20]. Among invasive fungal infections in humans, *Candida albicans* remains the most important cause and is associated with high morbidity and mortality [21]. As shown for *Candida*-induced sepsis in mice models, the fatal host damage results from an exaggerated immune response rather than from the pathogen itself [22]. Therefore, modulation of the immune response might be an interesting strategy to reduce *C. albicans*-associated immunopathology.

The orchestration of the antifungal response starts with the recognition of the pathogen by immune cells provided with fungal pattern recognition receptors (PRR) [23]. Monocytes express most of the fungal PRRs and have shown to play a particularly important role in the early recognition of the pathogen in invasive candidiasis [23, 24]. Moreover, monocytes are the most effective mononuclear cell-type at killing *C. albicans*, while their cytokine secretion is important for subsequent innate and adaptive immune activation [23, 25]. However, inflammatory monocytes have also been linked to dysregulated immune responses in invasive candidiasis [22]. Thus, modulation of the immune response in monocytes might be of particular importance for the course of infection. Among the PRRs expressed on these innate cells, Dectin-1 has shown to play a prominent role in the immune response against *C. albicans*, not only mediating phagocytosis but also triggering the oxidative burst and the production of several pro-inflammatory cytokines [26]. Hence, in the present study, we analyzed the immunomodulatory role of vitamin A on the innate immune response against *C.* *albicans* with a main focus on the Dectin-1-mediated response. For this purpose, we employed β-1,3-glucan beads which were designed to serve as “fungal-like particles” eliciting a dominant Dectin-1 response [27, 28].

## Materials and methods

### Material

Beta-1,3-glucan beads were prepared as previously described [27]. atRA was purchased from Sigma-Aldrich (Germany) and dissolved in absolute ethanol. The RARα-agonist BMS753, the RARγ-agonist BMS961, as well as the RARα antagonist BMS195614 and the RARγ antagonist MM11253 were purchased from Tocris Bioscience (UK). Monoclonal mouse anti-human Dectin-1 MAB1859 (clone #259931) antibody was purchased from R&D Systems (Germany). Mouse IgG_2B_ isotype control antibody was purchased from eBioscience (UK). APC-conjugated polyclonal goat anti-mouse antibody and APC-conjugated monoclonal mouse anti-human CD14 antibody were purchased from BD Biosciences (Germany). Polyclonal rabbit anti-Galectin-3 SC-20157 antibody was purchased from Santa Cruz (USA) and polyclonal rabbit anti-Actin (20–33) antibody was purchased from Sigma-Aldrich (Germany). HRP conjugated goat anti-rabbit IgG (H+L) antibody was purchased from Dianova (Germany).

### *Candida albicans* isolate

Overnight fungal cultures of the virulent wild-type strain SC5314 [29] were grown in YPD medium, washed three times and resuspended in PBS at a concentration of 10^8^ yeasts/ml. To avoid overbalanced growth of *C. albicans* and monocyte-killing due to hyphae formation, we inactivated the fungal yeasts. UV inactivation of the cells was performed on a UVC-500-Crosslinker (Amersham, UK) using two doses of 100,000 μj/cm^2^ immediately before cell stimulation.

### Monocyte isolation

Human monocytes were isolated from buffy coats kindly provided by Dagmar Barz (Institute of Transfusional Medicine of the Jena University Hospital). Peripheral blood mononuclear cells (PBMCs) were isolated by density gradient centrifugation following manufacturer’s instructions. Briefly, blood diluted 1:1 with PBS was layered onto an equal volume of Ficoll-Paque Plus (GE-Healthcare, Germany) and centrifuged in Leukosep Falcon tubes at 800 × g for 15 min. After centrifugation, the leukocyte band was collected, washed with cold NaCl 0.45 % and subjected to erythrocyte lysis using a hypotonic buffer. Cells were then washed twice in cold PBS and counted on a hemocytometer. Cell viability was assessed by trypan blue and propidium iodide/AnnexinV staining. To further isolate the monocytes, we used the monocyte isolation kit II (Miltenyi, UK) which couples negative selection with a cocktail of biotin-conjugated monoclonal antibodies and magnetic cell sorting using the quadro-MACS (Miltenyi, UK). Purity of the obtained monocytes was >92 % as assessed by CD14-labeling and flow cytometric analysis.

### Stimulation assays

After monocyte isolation, cells were resuspended at 4 × 10^6^ cells/ml in RPMI GlutaMax-Medium (Invitrogen, UK) supplemented with 1 % Penicillin/Streptomycin (Invitrogen, UK), plated on 6-well plates (VWR International, Germany) and allowed to equilibrate at 37 °C for 2 h. Monocytes were then pre-incubated with 1 μM of atRA or the specific RAR agonists for 30 min, followed by addition of the previously prepared *C. albicans* yeast at a fungus-monocyte ratio of 1:1. This ratio was predetermined in pilot experiments to preserve cell viability while yielding a suitable host gene response. When RAR antagonists were used, these were added 30 min before atRA, at a concentration of 1 μM. In the stimulation assay using β-1,3-glucan beads as specific ligands of Dectin-1, a 5:1 ratio was used. The cells were then incubated for 5 or 16 h at 37 °C and 5 % CO_2_. Viability of the monocytes was >90 %, as assessed by trypan blue and propidium iodide-staining. Additionally, AnnexinV staining was used to exclude an increase in apoptotic events. After incubation, the monocytes were harvested for RNA isolation and the culture supernatants were collected and stored at −80 °C.

### RT-PCR and quantitative PCR

To analyze the gene expression of the target genes, total RNA was isolated from 8 × 10^6^ monocytes using the Qiagen RNeasy mini kit. An additional step was included to remove the residual genomic DNA using DNaseI (Qiagen, Germany). A NanoDrop D-1000 Spectrophotometer (Thermo-Fisher Scientific, Germany) was then used to assess the amount and quality of the RNA. Complementary DNA (cDNA) was synthesized from 1,5 μg of RNA using the High Capacity cDNA Reverse Transcription Kit (Applied Biosystems, UK) following manufacturer’s instructions. For PCR-analysis, specific primers for each target gene were designed using the online primer-BLAST tool of the National Center for Biotechnology Information (NCBI, http://www.ncbi.nlm.nih.gov/tools/primer-blast/). In order to improve the PCR efficiency, possible secondary structures of the amplicons were taken into account by characterizing their nucleotide sequence using the Mfold algorithm [30]. The sequences of all primers used for amplification are listed in Table 1.

**Table 1:** Sequence of forward and reverse primers of indicated target genes and the size expected for each PCR product

Human gene	Symbol	Forward primer	Reverse primer	Size (bp)
Peptidylpropyl isomerase B	*PPIB*	ATGTAGGCCGGGTGATCTTT	TGAAGTTCTCATCGGGGAAG	219
Hypoxanthine phosphoribosyltransferase 1	*HPRT1*	GACCAGTCAACAGGGGACAT	AACACTTCGTGGGGTCCTTTTC	195
Tumor necrosis factor alpha	*TNFα*	TTCTCCTTCCTGATCGTGGC	ACTCGGGGTTCGAGAAGATG	150
Interleukin 6	*IL6*	GAGGAGACTTGCCTGGTGAA	TGGGTCAGGGGTGGTTATTG	186
Interleukin 10	*IL10*	GCTGAGAACCAAGACCCAGA	GCATTCTTCACCTGCTCCAC	143
Interleukin 12 subunit beta	*IL12B*	ACAACATCTGTTTCAGGGCCA	GGTCCAAGGTCCAGGTGATA	239
Dectin-1	*CLEC7A*	ACACTTCGACTCTCAAAGCA	TACAGCAATGAGGCGCCAA	91
Toll-like receptor 2	*TLR2*	TGCATTCCCAAGACACTGGA	AGGGAGGCATCTGGTAGAGT	131
Galectin-3	*LGALS3*	CCCATCTTCTGGACAGCCAA	CTTCACCGTGCCCAGAATTG	151
Retinoic acid receptor alpha	*RARA*	CCACATGTTCCCCAAGATGC	GCCCTCTGAGTTCTCCAACA	145
Retinoic acid receptor beta	*RARB*	TCGTCTGCCAGGACAAATCA	TTGGCATCGATTCCTGGTGA	158
Retinoic acid receptor gamma	*RARG*	CAAGGTCAGCAAAGCCCATC	ACTTGGTAGCCAGCTCACTG	137

PCR of the cDNA was carried out on a S1000™ Thermal Cycler (BioRad, UK) in a 25 μl reaction volume containing 0.2 μM primers, 1 U Taq DNA polymerase (5-Prime, UK) and 200 μM dNTPs. Thermal conditions included an initial 95 °C denaturation step for 3 min, and then 35 cycles of 10 s at 94 °C, 30 s at 60 °C and 30 s at 72 °C. The resulting PCR products were separated on an ethidium bromide stained agarose gel and visualized under a UV-transiluminator to confirm the expected amplicon size.

To quantify the relative expression of each gene, a Corbett Rotor-Gene 6000 (Qiagen, Germany) was used as Real-Time qPCR apparatus. Each sample was analyzed in duplicate in a total reaction volume of 20 μl containing 10 μl of 2 × SensiMix SYBR Master Mix (Bioline, UK) and 0.2 μM of each primer. All qPCRs were set up using a *CAS*-*1200 pipetting robot* (Qiagen, Germany). The cycling conditions were 95 °C for 10 min followed by 40 cycles of 95 °C for 15 s, 60 °C for 20 s and 72 °C for 20 s. For each experiment, an RT-negative sample was included as control. Specificity of the qPCRs was assessed by melting curve analysis and size verification by electrophoresis. The relative expression of the target genes was analyzed using a modified Pfaffl method [31, 32]. To determine significant differences in the mRNA expression between different experimental conditions, the relative quantity (RQ) for each sample was calculated using the formula 1/E^Ct^, where *E* is the efficiency and Ct the threshold cycle. The RQ was then normalized to the geometric mean of two housekeeping genes: hypoxanthine phosphoribosyltransferase1 (HPRT1) and peptidylpropyl isomerase B (PPIB). The stability of the housekeeping genes was assessed using the BestKeeper algorithm [33]. The normalized RQ (NRQ) values were log2-transformed for further statistical analysis with GraphPad PRISM v5.0.

### Flow cytometry

To analyze the expression of Dectin-1 on the cell surface, monocytes were washed with PBS containing 10 % FBS and stained with anti-Dectin-1 antibody (1 µg/ml, 30 min) and with APC-conjugated goat anti-mouse IgG (2,5 µg/ml, 30 min). A mouse-IgG_2B_-antibody was used as isotype control. Samples were measured on a FACSAria II apparatus (BD Biosciences; Germany) and data were analyzed using the FLOWJO 7.6.4 software. The resulting mean fluorescence intensities (MFIs) were normalized to those of unstained cells in each case.

### Cytokine measurements

TNFα, IL6, IL12 and IL10 secretion by the monocytes was analyzed using commercially available ELISA kits (Human TNFα Elisa Kit, Thermo Scientific; Human IL12 Elisa Kit, Hölzel Diagnostika; Human IL6 Elisa Kit, eBioscience; Human IL10 Elisa Kit, eBioscience) according to the manufacturer’s instructions. An Infinite M200 reader (Tecan, UK) was used to measure the optical density (OD), and the concentration of each cytokine was calculated from the respective standard curve by five-parameter logistic analysis using the Magellan v.6 software (Tecan, UK).

### Statistical analysis

Statistical analyses were performed using GraphPad PRISM v5.0 software (San Diego, USA). Two-sided pairwise *t* test or repeated measures ANOVA with Dunnett’s post hoc test were used to determine statistical significance. In all cases, the level of significance was set at *p* ≤ 0.05.

## Results

### Retinoic acid modulates the cytokine production induced by *C. albicans* and β-1,3-glucan in human monocytes

We assessed the impact of vitamin A on the immune response to *C. albicans* by challenging monocytes with UV-killed yeasts in the absence or presence of 1 μM atRA. In a first approach, we analyzed the expression of TNFα, IL6, IL12b and IL10 at transcriptional level by Real-Time qPCR. For accurate normalization purposes, we tested the stability of the housekeeping genes among the experimental conditions using the BestKeeper algorithm [33]. Since both PPIB (standard deviation (SD) of the Ct = 0,48; coefficient of variance (CV) of the Ct = 2,60) and HPRT1 (SD(Ct) = 0,49; CV(Ct) = 2,22) showed a highly stable expression, the geometric mean of both genes was used for further relative expression calculations.

**Fig. 1:**
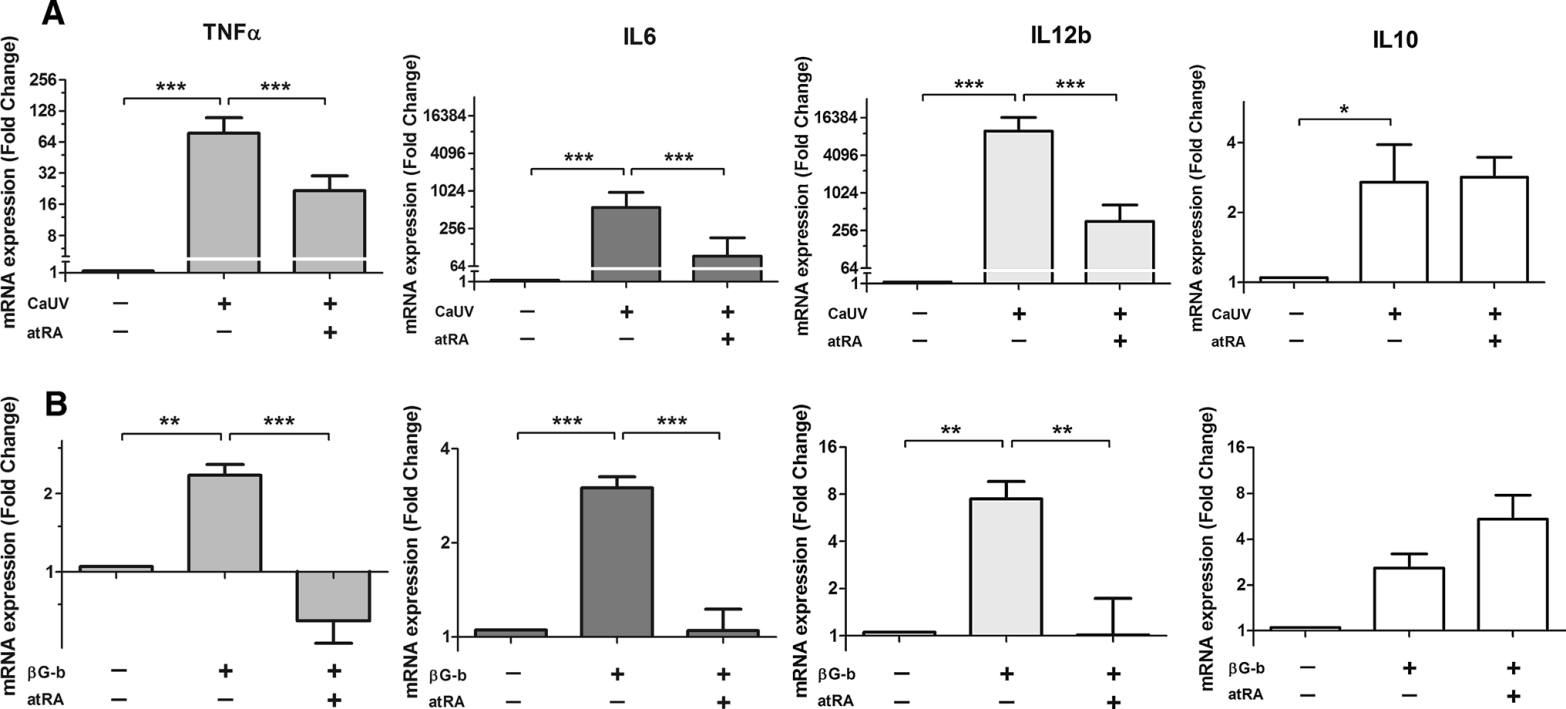
Relative mRNA expression levels of TNFα, IL6 and IL12b measured by qPCR. Monocytes were stimulated with either **a** UV-treated *C. albicans* yeasts (UV-Ca) or **b** β-1,3-glucan beads (βG-b) for 5 h in the presence or absence of 1 μM atRA. Both the β-1,3-glucan-induced and the *C. albicans*-mediated up-regulation of pro-inflammatory cytokine expression was significantly attenuated by atRA. Data were obtained from five independent experiments, each performed with cells from different donors. Results are presented as mean ± SEM of the fold change relative to the control (unstimulated cells). *** *p* ≤ 0.001

After 5 h of incubation with *C. albicans* yeasts, we could observe a clear increase in the mRNA expression levels of all four cytokines analyzed (Fig. 1a). However, in the presence of atRA in the cell culture medium, the up-regulation of the pro-inflammatory cytokines was significantly suppressed. As shown in Fig. 1a, the *C. albicans*-mediated expression of TNFα could be dropped from a 78-fold expression (in the absence of atRA) to a 21-fold expression when atRA was present. In a similar way, we could observe a significant reduction of 83 and 96 % in the gene expression levels of IL6 and IL12b, respectively (Fig. 1a). This modulation by retinoic acid occurs in a dose-dependent manner, and an inhibitory effect of atRA on all three cytokines could already be observed with concentrations as low as 0,01 μM (Suppl. Fig. 1). On the other hand, we could not observe any effect of atRA on the *C. albicans*-induced IL10 expression (Fig. 1a), even if in the absence of fungal challenge, the addition of atRA led to an up-regulation of this anti-inflammatory cytokine (data not shown).

Comparable results were obtained when the monocytes were stimulated with β-1,3-glucan beads to specifically address the Dectin-1-response (Fig. 1b). As shown in Fig. 1b, the activation of this early fungal-recognition receptor, leading to an up-regulation of all three pro-inflammatory cytokines, was down-regulated by atRA at transcriptional level. In contrast, the expression of the anti-inflammatory cytokine IL10 was rather potentiated by atRA, although not in a statistically significant manner.

**Fig. 2:**
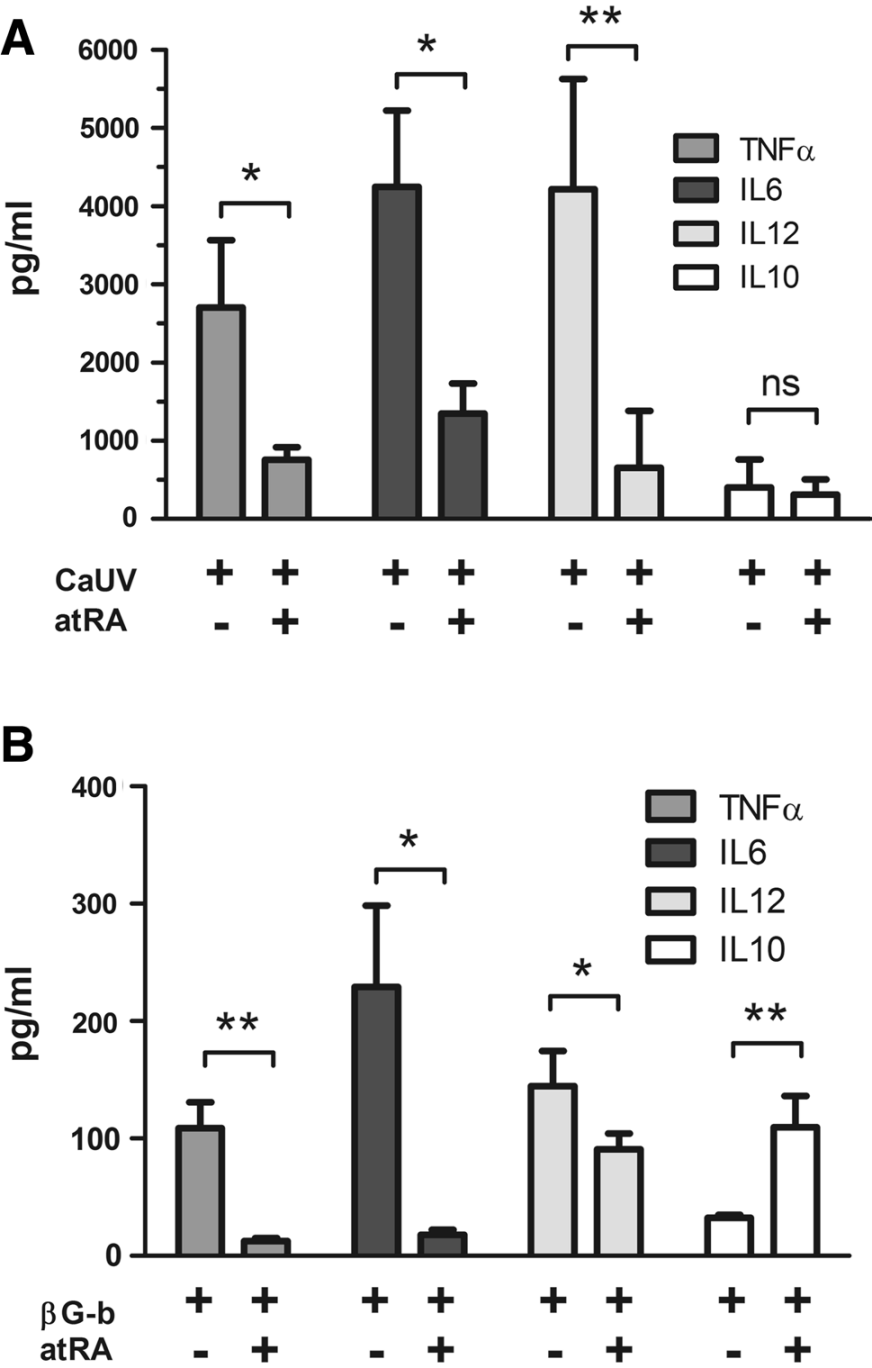
Cytokine measurement in the culture supernatants. Monocytes were stimulated with either **a** UV-treated *C. albicans* yeasts (UV-Ca) or **b** β-1,3-glucan beads (βG-b) in the presence or absence of 1 μM atRA. After 16 h culture supernatants were analyzed using specific ELISAs for TNFα, IL6, IL12 and IL10. In the presence of atRA, the secretion of all pro-inflammatory cytokines was significantly inhibited. Columns and *error bars* represent the mean ± SEM of five independent experiments. * *p* ≤ 0.05; ** *p* ≤ 0.01

To further confirm our findings at protein level, we analyzed the culture supernatants for the presence of TNFα, IL6, IL12 and IL10 after 16 h of stimulation with either *C. albicans* or β-glucan beads. As shown in Fig. 2, pro-inflammatory cytokine secretion upon fungal stimulation was severely affected by atRA. Co-stimulation with atRA decreased the amount of secreted TNFα, IL6 and IL12 in a significant manner, whereas no effect could be observed on the IL10 release. (Fig. 2a). Similar results were observed when the monocytes were stimulated with the β-glucan beads. In this case, atRA showed an inhibitory effect on the release of all pro-inflammatory cytokines, whereas the secretion of IL10 was rather potentiated (Fig. 2b). These observations resemble the results obtained at transcriptional level and suggest a strong anti-inflammatory role of vitamin A in fungal infections.

### Retinoic acid modulates the expression of Dectin-1

**Fig. 3:**
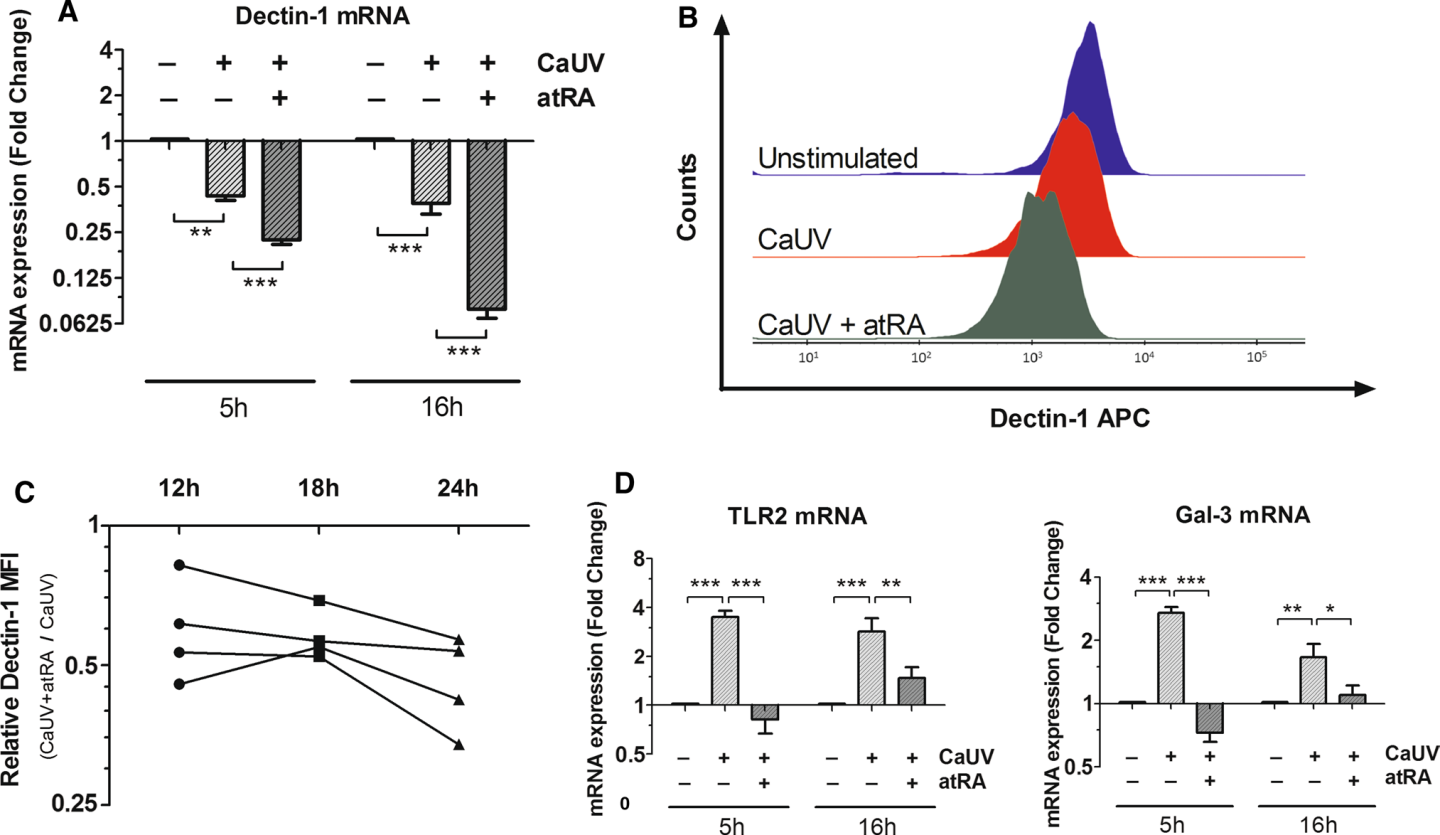
Modulation of the Dectin-1-expression by retinoic acid upon *C. albicans* infection. Monocytes were stimulated with UV-treated *C. albicans* (CaUV) yeasts in the presence or absence of 1 μM atRA. **a** Dectin-1-mRNA expression was measured after 5 and 16 h. **b** Representative flow cytometry plot of Dectin-1-APC after 24 h of stimulation. **c** Relative Dectin-1 MFI over time. The relative MFI is defined as the MFI of the cells challenged with *C. albicans* in the presence of atRA divided by the MFI of the monocytes challenged with the fungi in the absence of atRA. **d** mRNA expression of Dectin-1 co-receptors TLR2 and Galectin-3 after 5 and 16 h of stimulation. Data for the transcriptional analysis were obtained from five independent experiments, each performed with cells from different donors. Results are presented as mean ± SEM of the fold change relative to the control (unstimulated cells). For flow cytometry analysis, data from four independent experiments were collected. * *p* ≤ 0.05; ** *p* ≤ 0.01; *** *p* ≤ 0.001

Since we could observe an impact of vitamin A on the function of Dectin-1 in terms of the receptor-mediated cytokine production, we next wanted to investigate whether vitamin A had an effect on the expression of this PRR. Under the experimental settings of our stimulation assay, the expression of Dectin-1 was measured at transcriptional level after 5 and 16 h of incubation with *C. albicans*. At both time points, atRA supplementation led to a decreased expression of Dectin-1 mRNA. Moreover, the inhibitory effect of atRA seemed to increase over time, reaching a fivefold down-regulation of Dectin-1 mRNA after 16 h of incubation with *C. albicans* (Fig. 3a). Similar down-regulation was also observed when cells were stimulated with atRA alone, in the absence of fungal challenge (Suppl. Fig. 2). Interestingly, *C. albicans* itself was also able to slightly dampen the expression of Dectin-1 at transcriptional level (Fig. 3a).

Next, we explored the expression of Dectin-1 on the cell surface of the monocytes. As shown in Fig. 3b, our findings at transcriptional level could be reproduced at protein level by flow cytometry. A shift in the MFI became apparent after *C. albicans* stimulation and reached its maximum drop in the presence of atRA (Fig. 3b). To further assess the impact of atRA on Dectin-1 over time, relative MFI values were calculated for each time point. In agreement with the observations at transcriptional level, the atRA-mediated down-regulation of Dectin-1 increased over time as assessed by a trend test (Page’s *L* test; *p* < 0.001; Fig. 3c). These results suggest a sustained modulation of the anti-fungal response by vitamin A. Moreover, when we analyzed the influence of atRA on the expression pattern of two known co-receptors of Dectin-1, TLR2 and Galectin-3, a highly significant down-regulation could be observed in both cases (Fig. 3d). While *C. albicans* increased the mRNA expression of both TLR2 and Galectin-3, this up-regulation was almost completely abrogated in the presence of atRA.

### RAR-dependent and RAR-independent mechanisms mediate the atRA-induced anti-inflammatory effect in monocytes

**Fig. 4:**
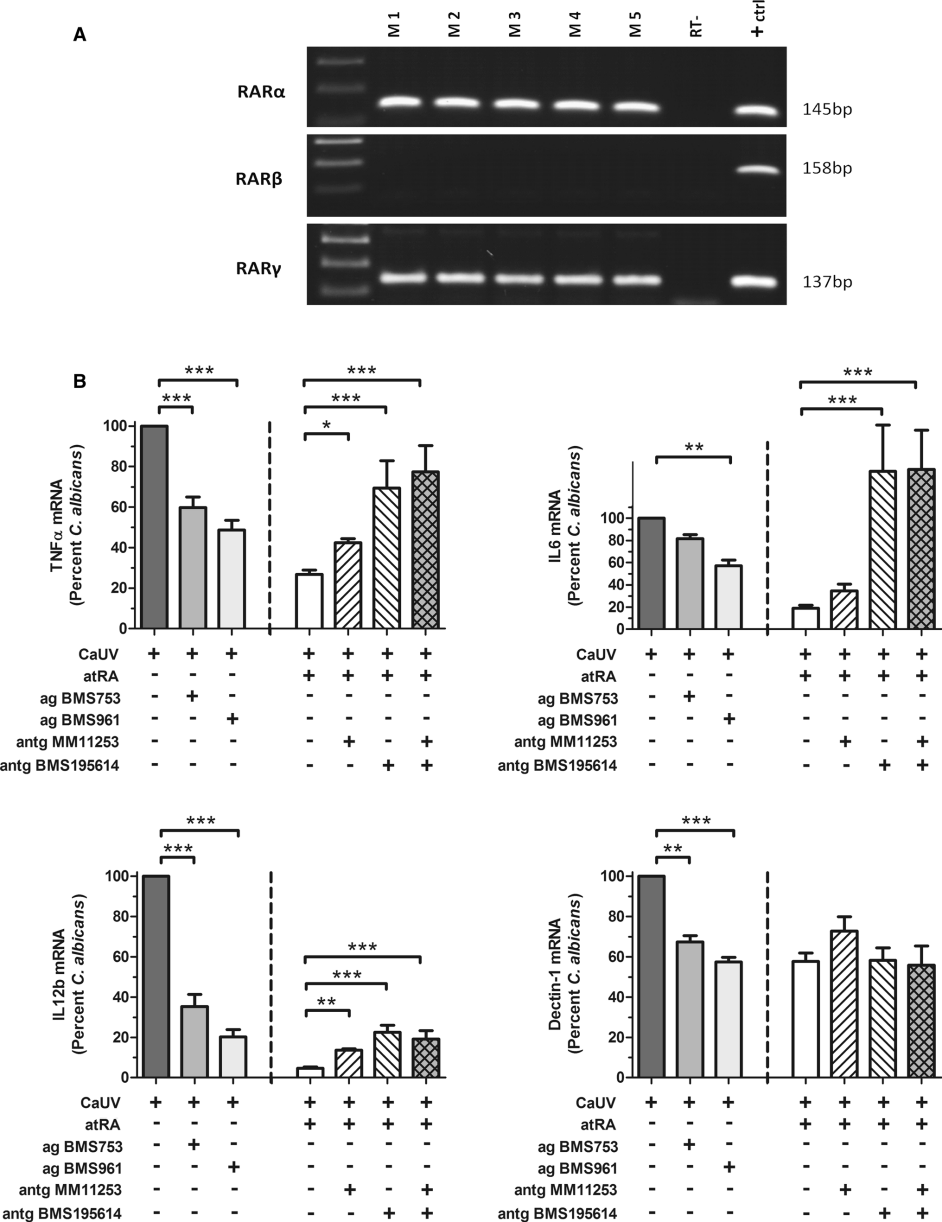
Expression profile and immunomodulatory activity of retinoic acid receptors in human monocytes. **a** Transcriptional expression of RARα, RARβ and RARγ in monocytes (samples M1-M5) as discriminated by agarose gel electrophoresis of PCR products. HEK293 cells (RARα), A549 cells (RARβ) or NHBE cells (RARγ) served as positive controls in each case. (RT- = reverse transcription negative control) **b** Immunomodulatory effect of specific RAR-agonists BMS753 and BMS961 on the expression of TNFα, IL6, IL12b and Dectin-1 upon *C. albicans* infection. Also shown is the inhibitory power of the specific RAR antagonists MM11253 and BMS195614 on the atRA-mediated modulation of these genes. Monocytes were pre-incubated for 30 min with either RAR-specific agonists or atRA (in the presence or absence of RAR antagonists) and then challenged with UV-treated *C. albicans* yeasts (CaUV). Columns represent the percentage of the *C. albicans*-induced mRNA expression in each case. Data were collected from five independent experiments. * p ≤ 0.05; ** *p* ≤ 0.01; *** *p* ≤ 0.001

To determine the involvement of specific nuclear receptors in our findings, we first characterized the expression profiles of all possible RARs in our monocytes by RT-PCR. We could not detect any expression of RARβ, but obtained a clear signal for the expression of both RARα and RARγ mRNA in all five monocyte samples used in this study (Fig. 4a). To define the relevance of these two receptors in the atRA-mediated anti-inflammatory effect, specific agonists and antagonists of each RAR were used in our experimental setting.

When the monocytes were pre-incubated with the specific RAR agonists, both RARα agonist BMS753 and RARγ agonist BMS961 were able to mimic the repressive effect of atRA, although only partially in most cases (Fig. 4b). These results suggest that both RAR receptors are capable to mediate the observed down-regulation of the inflammatory response against *C. albicans*. To further prove their involvement in our particular experimental setting, specific antagonists of both RARs were used to test whether the expression levels of each gene could be restored in their presence. For both TNFα and IL6, the *C. albicans*-induced expression levels could be fully restored in the presence of the RAR antagonists in a significant manner (Fig. 4b). Also the expression of IL12b could be restored significantly, but only partially. In contrast, the atRA-mediated down-regulation of Dectin-1 mRNA was not affected by the addition of the RAR antagonists (Fig. 4b). Taken together, these results suggest a RAR-dependent mechanism for the atRA-mediated modulation of the pro-inflammatory cytokines, whereas an additional RAR-independent mechanism seems to play a major role in the transcriptional regulation of Dectin-1.

## Discussion

Despite the increasing interest in the immunomodulatory role of vitamin A, no evidence has been reported addressing the impact of vitamin A on the immune response to fungi. In the present study, we have characterized the effect of atRA on the *C. albicans*-induced immune response in human monocytes. Our results show a strong immunomodulatory role for atRA, leading to a highly significant suppression of the fungi-induced expression of TNFα, IL6 and IL12. This down-regulation could be assessed at both transcriptional and post-translational level.

In fungal infections, the immune response is initiated after recognition of the pathogen by specific PRRs, such as Dectin-1 [26]. To address the impact of vitamin A on this important receptor in the fungal immune activation, we stimulated the monocytes using β-1,3-glucan beads as specific Dectin-1 ligand. Under these conditions, the modulatory effect of atRA resembled the one observed in experiments performed with *C. albicans*. Besides the impact of atRA on the functional output of Dectin-1 activation, we investigated the effect of atRA on the expression of the receptor upon *C. albicans* challenge. Interestingly, *C. albicans* alone was able to down-regulate the expression of Dectin-1, which has not been described previously in the literature. This observation might suggest a new mechanism to be included in the growing record of immune-escaping strategies described for this fungus [34, 35]. When atRA was added in this experimental setting, an even stronger inhibitory impact on the expression of Dectin-1 could be observed. The atRA-mediated down-regulation of the receptor could be assessed at transcriptional level and by flow cytometry. The inhibitory effect of atRA was not challenge-dependent, since a similar effect could be observed in the absence of *C. albicans* (Suppl. Fig. 2). This effect seems also not to be related to a process of atRA-induced differentiation, since a comparable down-regulation of Dectin-1 could be observed when terminally differentiated dendritic cells were stimulated with atRA (data not shown). Moreover, we could demonstrate that the suppressive effect of atRA increased over time, which suggests that the immunomodulatory impact of vitamin A might be sustained over a prolonged period. In addition, the known Dectin-1 co-receptors TLR2 [36] and Galectin-3 [37], which have been shown to enhance the Dectin-1-dependent immune response, were also down-regulated by atRA.

Our results raised the question whether the observed drop in pro-inflammatory cytokine production could be explained by the atRA-mediated down-regulation of Dectin-1 and its co-receptors. The effect of atRA on the cytokine expression could be observed at transcriptional level after 5 h of incubation. Interestingly, at this stage, the atRA-mediated Dectin-1 down-regulation at the cell surface was already apparent, although only in a very incipient manner (Suppl. Fig. 3). Therefore, we cannot exclude that the down-regulation of the receptor might contribute, at least in part, to the observed drop in cytokine levels. Nevertheless, it is likely that other direct mechanisms play a prominent role in the early phase of the atRA-mediated immunomodulation. This is supported by our observation that short pre-incubation periods with atRA led to a stronger down-regulation of the fungi-induced cytokine expression than pre-incubation periods of 24 h, when the Dectin-1-down-regulation on the cell surface reached a maximum (Suppl. Fig. 4). This is also in agreement with the observation that atRA has been described to modulate the immune response to LPS, a Dectin-1-independent immunological challenge, at least in other cell types such as dendritic cells and macrophages [16].

In monocytes, the immunomodulatory role of atRA on the immune response against LPS has been reported by Oeth et al. [38] and Wang et al. [39]. While Oeth et al. could not detect any atRA-mediated changes in the TNFα secretion after stimulation with LPS, Wang et al. observed only a very slight impact of atRA (<twofold change) on the LPS-induced TNFα and IL12 mRNA expression levels. In our study, the addition of atRA led to an almost 100 % abrogation of the Dectin-1-mediated expression and secretion of TNFα, IL6 and IL12 in human monocytes. It would be interesting to investigate whether the higher impact of atRA observed in our study could be related to the nature of the immunological challenge. The signal transduction of the LPS/TLR4 axis differs consistently from that of the Dectin-1-activation [26]. It is tempting to speculate that different PRR-signaling pathways might display different sensitivities to atRA-modulation. Further studies are required to comprehensively elucidate the mechanisms of atRA-mediated modulation of the immune response.

Most of the biological actions of atRA are exerted through binding its specific nuclear receptors: RARα, RARβ and/or RARγ [40]. We investigated the expression of all three RARs in monocytes and could detect the expression of both RARα and RARγ mRNA, which is in agreement with previous studies [41]. The use of specific agonists and antagonists for each RAR allowed us to verify that both receptors are involved in the suppressive effects observed on TNFα, IL6 and IL12. On the other hand, the vitamin A modulation of the Dectin-1 expression seemed to occur mainly through a RAR-independent mechanism.

The impact of vitamins on immunity and inflammation has been widely investigated in allergy and bacterial infections [42], but to a much lesser extent in fungal infections. Nevertheless, vitamin D has already been described to possess significant anti-inflammatory properties in *Candida* infections [43]. Meanwhile, the role of vitamin A in invasive candidiasis has remained unknown. Moreover, only very few studies have been conducted to investigate the vitamin A-status in septic patients, were *C. albicans* is a common nosocomial pathogen [44, 45]. Indeed, 33–55 % of all episodes of candidemia have been shown to occur in intensive care units (ICU) [46]. Such ICU-acquired candidemia in critically ill patients is associated with a particular high mortality of >50 % [47]. The search for anti-inflammatory immunomodulators is of particular importance in invasive candidiasis, where the dysregulation of the immune response rather than the pathogen has been shown to be responsible for the fatal host damage [22, 43]. Interestingly, a recent study reported an important inadequacy of retinol and β-carotene in a cohort of sepsis patients [45]. Thus, monitoring the serum levels of vitamin A and its adequate supplementation in individuals admitted in ICUs might have far-reaching prophylactic implications.

In conclusion, in this study, we have demonstrated a strong immunomodulatory role of vitamin A on the innate host response to *C. albicans* in human monocytes. In addition, we could show that atRA modulates both the function and the expression of Dectin-1. Moreover, the modulation of this PRR seems to increase over time, leading to a sustained regulation of the immune response. The observed effect of atRA on the cytokine production is likely to occur via activation of either or both RARα and RARγ. This study opens a new avenue to explore the role of vitamin A in fungal infections and to elucidate further molecular mechanisms of its immunomodulatory function.

## Electronic supplementary material

**Below is the link to the electronic supplementary material.**

**Suppl. Figure** **1.- Dose-dependent regulation of the cytokine mRNA expression by retinoic acid.** The modulatory effect of increasing concentrations of atRA (0.01 µM – 10 µM) on the *C. albicans*-induced cytokine expression was measured by qPCR. Shown is the percentage of inhibition as normalized to the *C. albicans*-induced mRNA expression of each gene. **(TIFF 22,313** **kb)**

**Suppl. Figure** **2.- Modulatory role of atRA on the Dectin-1 expression in the absence of inflammatory stimuli.** We measured the effect of atRA on the expression of Dectin-1 A) at transcriptional level after 5 h of incubation with 1 µM atRA by qPCR, and B) at the cell surface after 24 h of incubation by flow cytometry (data representative of 3 biological replicates). * p ≤ 0.05 **(TIFF 67,138** **kb)**

**Suppl. Figure** **3.- Analysis of the expression of Dectin-1, TLR2 and Galectin-3 after 4** **h of incubation with** ***C. albicans*** **(in the presence or absence of 1** **µM atRA).** Receptor expression was measured on protein level by flow cytometry for A) Dectin-1 and B) TLR2, and by Western Blot for C) Galectin-3 after 4 h of incubation. Incipient regulation of the Dectin-1 expression is observed after *C. albicans* challenge, being potentiated in the presence of atRA. The data are representative of 3 independent experiments. **(TIFF 59,077** **kb)**

**Suppl. Figure** **4.- Regulation of the cytokine mRNA expression by retinoic acid exposure over different time-periods.** Monocytes were pre-incubated with 1 µM atRA for either 0,5 or 24 h. Then the cells were challenged with *C. albicans* for 5 h and the cytokine expression was measured by Real-Time qPCR. Shown is the percentage of inhibition achieved by atRA pre-incubation on the *C. albicans*-induced cytokine expression. **(TIFF 27,642** **kb)**
