# Supplementary figures and images for: Modulatory role of vitamin A on the Candida albicans-induced immune response in human monocytes
Source: Med Microbiol Immunol. 2014 Aug 17;203(6):415–24. doi: 10.1007/s00430-014-0351-4 (PMC4232755; doi:10.1007/s00430-014-0351-4)

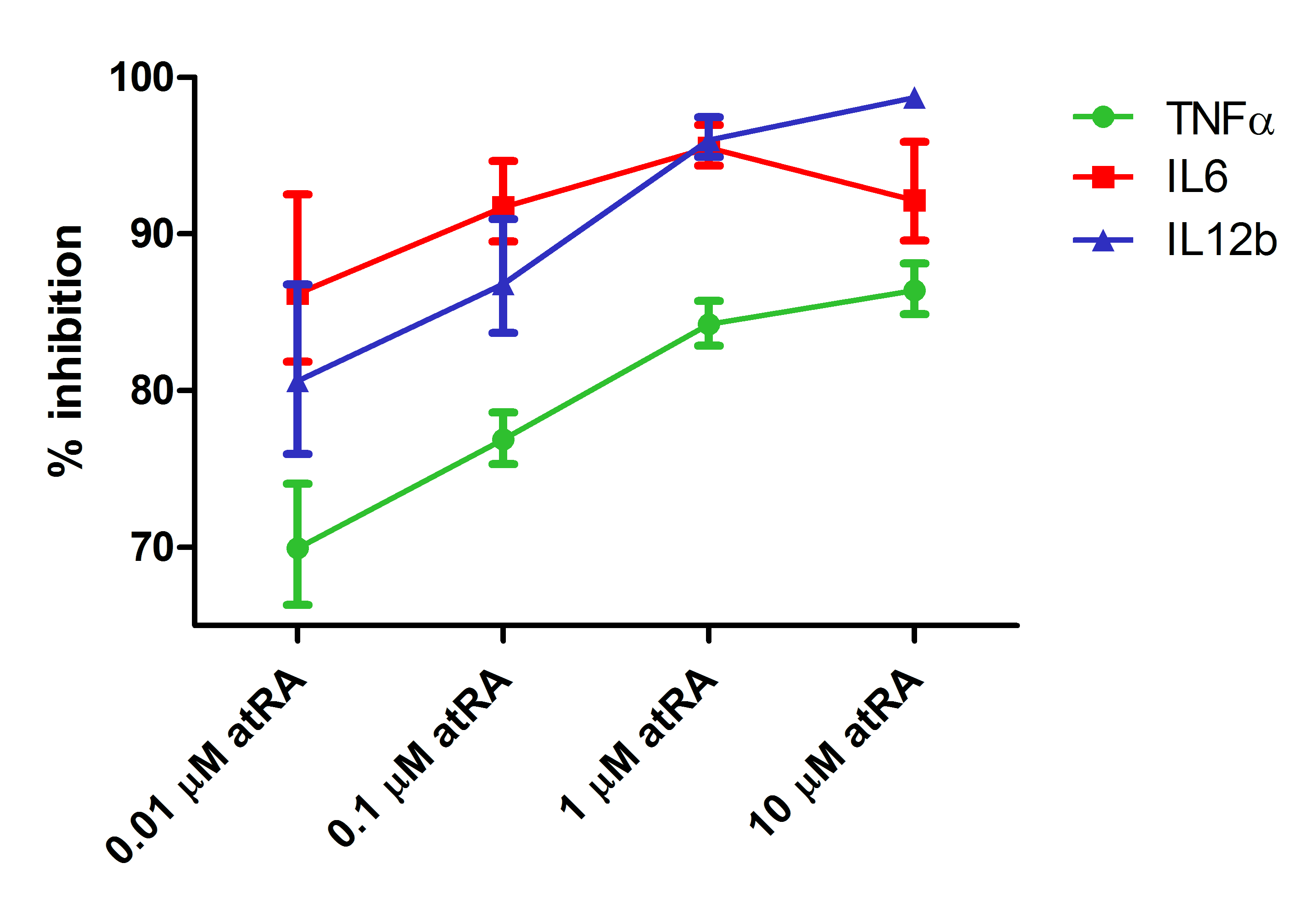

Supplement: Supplementary file 1 — Suppl. Figure 1.- Dose-dependent regulation of the cytokine mRNA expression by retinoic acid. The modulatory effect of increasing concentrations of atRA (0.01 µM – 10 µM) on the C. albicans-induced cytokine expression was measured by qPCR. Shown is the percentage of inhibition as normalized to the C. albicans-induced mRNA expression of each gene. (TIFF 22,313 kb) [file 430_2014_351_MOESM1_ESM.tif]

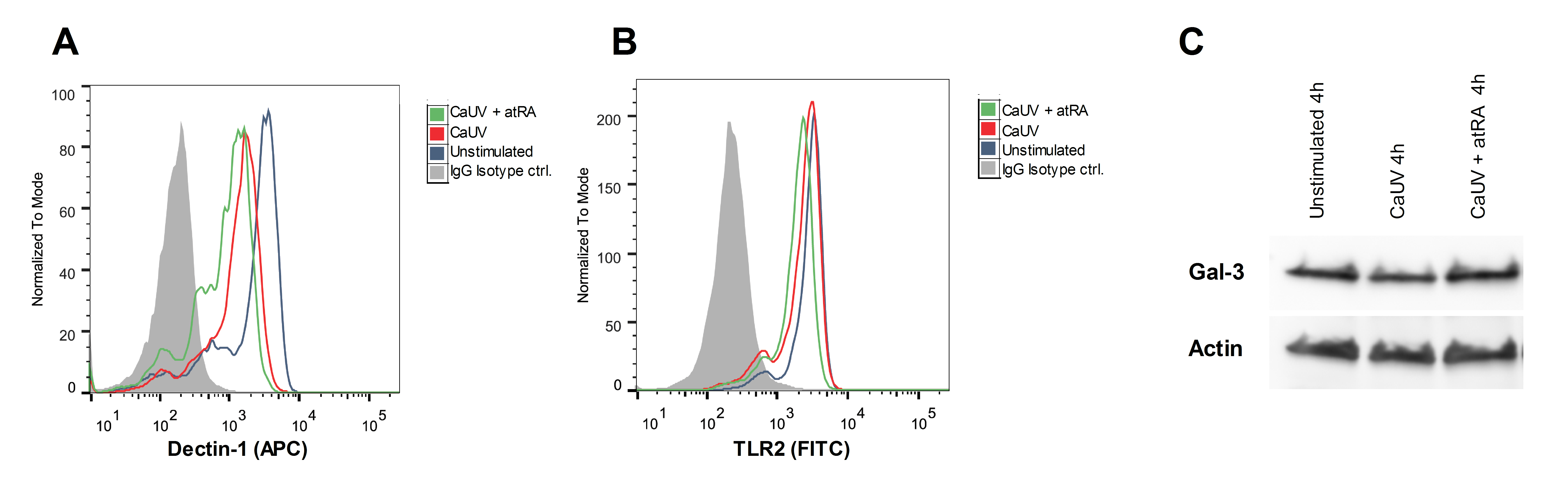

Supplement: Supplementary file 3 — Suppl. Figure 3.- Analysis of the expression of Dectin-1, TLR2 and Galectin-3 after 4 h of incubation with C. albicans (in the presence or absence of 1 µM atRA). Receptor expression was measured on protein level by flow cytometry for A) Dectin-1 and B) TLR2, and by Western Blot for C) Galectin-3 after 4 h of incubation. Incipient regulation of the Dectin-1 expression is observed after C. albicans challenge, being potentiated in the presence of atRA. The data are representative of 3 independent experiments. (TIFF 59,077 kb) [file 430_2014_351_MOESM3_ESM.tif]

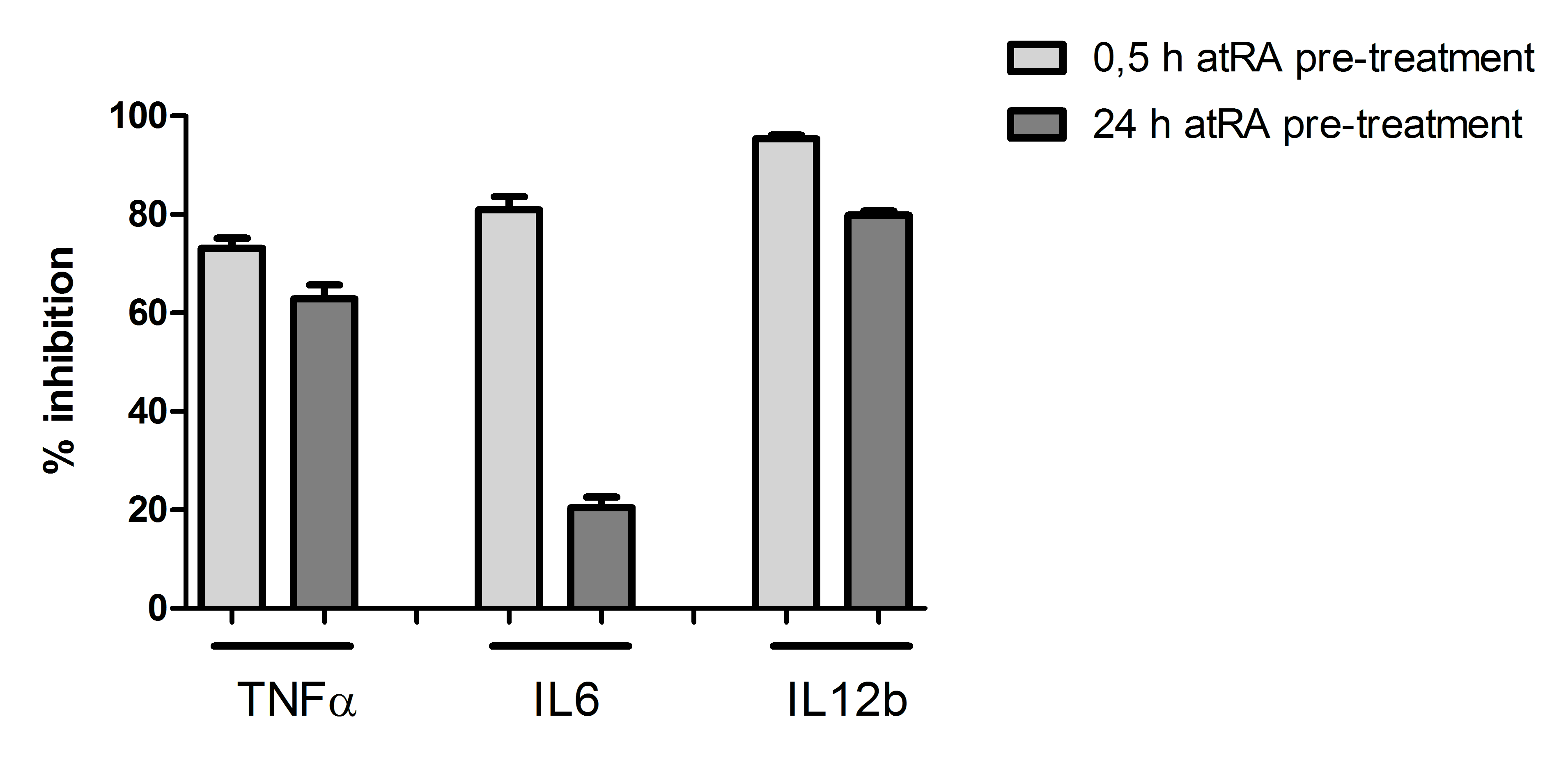

Supplement: Supplementary file 4 — Suppl. Figure 4.- Regulation of the cytokine mRNA expression by retinoic acid exposure over different time-periods. Monocytes were pre-incubated with 1 µM atRA for either 0,5 or 24 h. Then the cells were challenged with C. albicans for 5 h and the cytokine expression was measured by Real-Time qPCR. Shown is the percentage of inhibition achieved by atRA pre-incubation on the C. albicans-induced cytokine expression. (TIFF 27,642 kb) [file 430_2014_351_MOESM4_ESM.tif]
